# Retraction: Gut Microbiota Is a Key Modulator of Insulin Resistance in TLR 2 Knockout Mice

**DOI:** 10.1371/journal.pbio.1002479

**Published:** 2016-05-23

**Authors:** Andréa M. Caricilli, Paty K. Picardi, Lélia L. de Abreu, Mirian Ueno, Patrícia O. Prada, Eduardo R. Ropelle, Sandro Massao Hirabara, Ângela Castoldi, Pedro Vieira, Niels O. S. Camara, Rui Curi, José B. Carvalheira, Mário J. A. Saad

The authors and editors retract this publication following an investigation into concerns around the data presented in several figures that were brought to the editors’ attention. The text below has been agreed to by the editors, the first author (who was a PhD student at the time of publication), the corresponding author and most of the co-authors. All co-authors have been informed.

Concerns were raised about Figures 1, 4, 5, 6, 7, 8 and 10, and Figure S6. The authors state that although the data described were correctly obtained with high reproducibility, they inadvertently used the wrong blots in several figures resulting in duplications. The blots in question are Beta-Actin for Figures 1J, 5E, 6F, 7F, 8L, 10I, and S6A and C; IRS-1 for Figure 4G and I; Akt for Figure 7G; pJNK for Figure 4C, 10E and F; and IR for Figure S6B. In Figure 6C (Akt) and 6D (pJNK) one extra band was inadvertently included because the authors used an extra sample from the WT control mouse. In Figure 8J (Beta-Actin) there is one band missing. For Figures 10B and S6A upper panel, the authors did not indicate that the lanes 2 and 3 were non-contiguous. It should be noted specifically that the co-authors from collaborating groups (AC, PV & NOSC) were not involved in the preparation of these figures.

An institutional inquiry has been undertaken at the University of Campinas (UNICAMP) in São Paulo, Brazil and while the investigation acknowledged these errors, they did not find evidence of research misconduct. The authors maintain that the errors do not affect the interpretation of the results nor the conclusions of the study. However, the authors accept that the preparation of the figures fell below the standard of publication and therefore the authors and editors agree that the correct action is to retract the article. The authors apologize to the scientific community and will seek to publish a corrected manuscript version corroborating the findings of this work.
